# Exploring the Views of Osteogenesis Imperfecta Caregivers on Internet-Based Technologies: Qualitative Descriptive Study

**DOI:** 10.2196/15924

**Published:** 2019-12-18

**Authors:** Aimee R Castro, Khadidja Chougui, Claudette Bilodeau, Argerie Tsimicalis

**Affiliations:** 1 Ingram School of Nursing McGill University Montreal, QC Canada; 2 Shriners Hospitals for Children-Canada Montreal, QC Canada

**Keywords:** smartphone, caregivers, pediatrics, rare diseases, telemedicine, quality improvement, social media, chronic disease, osteogenesis imperfecta

## Abstract

**Background:**

Osteogenesis imperfecta (OI) is a rare genetic condition that can lead to frequent debilitating bone fractures. Family caregivers of children with OI face unique challenges in providing care, which may include limited access to information about the condition, feelings of distress, and experiences of social isolation. Internet-based technologies (IBTs) have been useful for supporting other types of caregivers. However, the views of OI caregivers on IBTs have not been explored.

**Objective:**

This study aimed to explore the views of OI caregivers on the uses of IBTs to support them in caring for their children with OI.

**Methods:**

A qualitative descriptive study was conducted. Caregivers of children with OI were recruited at a pediatric hospital in Montreal, Canada. Interviews were used to explore each caregiver’s views on the applicability of IBTs in supporting their caregiving needs. The interviews were transcribed verbatim and thematically analyzed.

**Results:**

A total of 18 caregivers participated. The caregivers shared that IBTs were useful for facilitating the following activities: daily activities of caregiving (such as providing physical care, supporting relationships, supporting self-care and hope, and managing the logistics of caregiving), OI medical information seeking, and OI social networking. However, they also revealed concerns about the health consequences of IBT use and the quality of IBT content. Concerns regarding IBTs varied somewhat with caregivers’ geographies. Caregivers offered suggestions and strategies for how IBTs can be optimized for caregiving.

**Conclusions:**

Family caregivers of children with OI face unique challenges in providing care, which may include lacking access to information about the rare condition and feeling socially isolated. OI caregivers use IBTs to overcome some of these challenges and to support their specific caregiving needs. These findings contribute to the paucity of knowledge by offering varied IBT strategies to support caregiving activities, which may be beneficial for other caregivers. Participants’ suggestions for IBT services can inform the development of new resources for OI caregivers and potentially for other caregivers of children living with rare conditions.

## Introduction

### Background

Osteogenesis imperfecta (OI) is a rare genetic condition (affecting 1 in 10,000 births) that alters collagen production [1,2]. OI is characterized by increased bone fragility and reduced bone mass, which increase the risk of fractures [1,2]. Although many OI types are distinguished in the genetic literature, the majority of individuals with OI are diagnosed with OI type I, which has the mildest disease severity [1]. OI type II is deadly in the neonatal period. OI type III is the most severe form of OI that is compatible with long-term survival. OI type IV is of moderate disease severity [1]. OI types V, VI, and VII are newer classifications that present with different bone and tissue phenotypes; these variants have a mild-to-moderate risk for bone fractures [1,2]. At present, there is no cure for OI; thus, ongoing and frequent physiotherapy, occupational therapy, surgery, and bisphosphonate medications remain to be the course of treatment [1,3,4].

### Caregiving and Osteogenesis Imperfecta

In chronic conditions such as OI, family caregivers (*caregivers*) play a critical role in providing safe, effective, and supportive care for patients [5]. For many caregivers, although positive experiences can result from raising a child with OI [3,6], the diagnosis itself is often “devastating” [3], leading to subsequent “ups and downs” [3] as families adjust to the diagnosis. The challenges faced by OI caregivers can include financial difficulties, transportation and accessibility challenges, a lack of knowledgeable health care providers, suspicions of child abuse leading to the diagnosis, social isolation, and a lack of social support [3,6-8].

### Caregiving and Internet-Based Technologies

Caregivers of patients with various other childhood- and adult-onset conditions have found that internet-based technologies (IBTs), such as websites, email, social media, mobile apps, and video calls, facilitate knowledge exchange and provide social support in their caregiving support needs [5,9-13]. Research on the uses of IBT for caregivers of children with special needs is an emerging field [13-15]. By understanding these caregivers’ views on IBTs, future platforms may be created to meet their unique needs [13,16]. As far as we are aware, no researchers have studied the uses of IBTs among caregivers of children with OI. Therefore, the aim of this study was to explore the views of caregivers of children with OI on the uses of IBTs in supporting their caregiving needs.

## Methods

### Design

A qualitative descriptive study [17] was conducted at a university-affiliated, pediatric orthopedic hospital in Montreal, Canada, where health care services are provided both in English and French to patients. Ethical approval was received from McGill University’s institutional review board (study number A05-B39-17B) and the study site before setup and recruitment.

### Recruitment

Purposive sampling was used to recruit adults (older than 18 years) who brought a patient (aged between 0 and 21 years) with OI to the study site for an OI-related appointment and who considered themselves to be a caregiver for the child. A nonauthoritative member of the team approached clinicians who had appointments with patients with OI to mediate an introduction to the study. If caregivers expressed an interest in learning more about the study, then a member of the research team described the study and informed the caregiver of the potential consequences of participating. If the caregiver provided their written consent to participate, then an interview was scheduled at the caregiver’s convenience.

### Data Collection

Each of the caregivers participated in 1 individual semistructured interview that took place in person at the hospital or by telephone or videoconferencing. All interviews were audio taped. Either the first author (AC) or the research assistant (KC) conducted the interviews by using an interview guide for caregivers who identified as IBT users (Multimedia Appendix 1). We prepared a second guide for interviewing caregivers who might not consider themselves to be regular users of IBTs; however, all caregivers who agreed to be interviewed identified as regular IBT users, so this second guide was never used. Demographic data were collected in a short demographic survey. Field notes were recorded after each interview.

### Data Analysis

Demographic data were analyzed using descriptive statistics in Microsoft Excel. Audio recordings were transcribed by the researchers who conducted the interviews (AC and KC). The French interviews were transcribed in French and open coded into English by KC, who is fluently bilingual in both English and French. The transcribed data and field notes were uploaded into Excel where they were open coded and thematically analyzed by AC, with additional intercoder verification by KC [18]. In regular meetings with the bilingual principal investigator (AT), the research team shared and discussed progress and interpretations of the data and the ongoing inductive development of the codebook [19]. These codebooks and meeting notes were recorded in a research journal, as were the ongoing research reflections of the first author (AC). The carefully anonymized research journal, larger samples of coded data, and the codebook may be made available for audits upon request.

To improve the trustworthiness of this qualitative research, we were guided by Lincoln and Guba’s [20] 4 criteria of credibility, dependability, confirmability, and transferability. The reflexive journaling by AC, the audio taping of interviews, and the research team’s regular peer debriefing enhanced the credibility of our research [21]. Carefully documenting our team’s discussions and decisions in our research journal and making the anonymized research journal, coding samples, and codebook available for audit enhanced the dependability of this study [21]. The carefully documented decision trail in our research journal and our frequent team debriefings supported the confirmability of this study [21]. The team development of our codebook and intercoder checks, our field notes, and our inclusion of thick descriptions and quotes support the transferability of our research [21].

## Results

### Demographic Data

A total of 18 adult caregivers from 14 families were interviewed for this study (Table 1). The interviews ranged from 15 min to 1.5 hours. These interviews resulted in 13.05 hours of audio recordings. Most of the caregivers (16/18) had the support of another adult providing care assistance at home. Functional abilities of their children ranged from having no need for any mobility assistance devices to using a wheelchair full time outside the home.

### Internet-Based Technology Views: Uses, Concerns, Strategies, and Suggestions

All families in our study had access to the internet at home. Most felt confident in their ability to navigate the internet and mobile apps. Furthermore, in their survey, 14 of the 18 participants responded that they had a generally positive view of using IBTs in relation to caring for their child with OI; in addition, 2 of them had a neutral view of IBTs and 2 had a negative view of IBTs. Caregivers described multiple uses of IBTs, reported some concerns, and shared their strategies and suggestions for optimizing future IBT development.

We used the following identification format to refer to specific participants throughout this paper: (Caregiver type) (Family number)-(OI type). Caregiver types were fathers (F), mothers (M), and legal guardians (G).

#### Uses of Internet-Based Technologies

##### Facilitating Daily Activities Related to Caregiving

###### Providing Physical Care

Caregivers described using IBTs to support physical caregiving activities by promoting rest and immobilization after fractures or surgeries and by being a tool for distracting their child. As a mother explained, “TV and the tablet is a hell of a distraction for pain” [M5-III]. For another family, the biggest challenge in caring for their 19-month old with OI was in trying to keep her immobilized for several weeks after her leg surgeries:

She got her [femur-rodding] surgery two weeks ago...she used to be able to crawl everywhere she wanted to...So, what we did most recently is, with the advice from a social worker here at [the study site] she said, “you know, you can have all these opinions about electronics, but you know, for those couple of weeks she’s going to be immobilized, we don’t need to feel bad about making her use electronics, or watching more TV than usual.” And that’s exactly what we did.

F1-IV

For these parents, Web-based videos and new computer tablet games provided calming distractions to help prevent their child from moving around when he or she needed to rest. In addition, caregivers found video streaming apps to be very helpful for occupying their children when the local environment was unsafe (eg, slippery mopped floors) and for distracting their children from pain after a fracture, during splinting, or during intravenous procedures.

**Table 1:** Demographic data were collected from the caregiver participants.

Demographic traits		Values^a^
Individuals interviewed (number of families represented)		18 (14)
**Relationship to child with OI^b^ (N=18 caregivers), n**		
	Mother	11
	Father	5
	Other: legal guardian	2 (same family)
**Language of interview (N=18 caregivers), n**		
	English	12
	French	6
Caregivers' median age in years (age range)		37.5 (24-57)
**Caregiver’s sex (N=18 caregivers), n**		
	Female	13
	Male	5
**Residential region (N=14 families), n**		
	Local (<2 hours’ drive from Montreal)	7
	Within Quebec, not local	1
	Other Canadian region	3
	International	3
**Highest level of education (N=18 caregivers), n**		
	Some postsecondary (university or college)	5
	Received university or college diploma	11
	Postgraduate	2
**Marital status (N=18 caregivers), n**		
	Married or common law	14
	Single (never married)	2
	Separated or divorced	2
**Estimated family income (N=14 families), n (Can $)**		
	<$25,000	3
	$25,000-$50,000	1
	$50,000-$80,000	1
	>$80,000	7
	Do not know	1
	Prefer not to answer	1
**Child’s OI type (N=14 families), n**		
	I	3
	III	4
	IV	6
	VI	1
**Ages of children with OI (17 children with OI), n**		
	Baby (0-12 months)	2
	Toddler (13 months-3 years)	1
	Preschool (4-5 years)	1
	School aged (6-12 years)	11
	Teenager (13-18 years)	2
**Family history of OI (N=14 families), n**		
	Yes	3
	No	11
**Internet access at home (N=14 families), n**		
	Yes	14
	No	0
**Comfort using the Internet (N=18 caregivers), n**		
	Very comfortable	14
	Somewhat comfortable	3
	Neutral	1
	Somewhat uncomfortable	0
	Uncomfortable	0
**General thoughts on using the internet for OI care (N=18 caregivers), n**		
	Positive	14
	Neutral	2
	Negative	2

^a^Values represent the number of individuals, unless otherwise indicated.

^b^OI: osteogenesis imperfecta.

###### Supporting Relationships

Caregivers also described how they used IBTs to bond with their children and extended families. A father explained how he used IBTs for low-impact activities, such as listening to audiobooks when he put his daughters to bed each night, doing Web-based yoga videos, and playing Pokémon Go in new cities for father-daughter dates. Traveling presented additional difficulties for families of children with fragile bones. One mother explained how her toddler stays connected with her grandparents through video calling even though those grandparents live farther away:

FaceTime with my parents—she actually does that a lot! “Mommy, mommy! I want to see Pappi!”...she asks for them. When my father’s not home to do that FaceTime, it’s like “Where is he?! Why?!”

M1-IV

For some families, visiting other individuals’ houses posed a fear of fractures being caused by furniture or wobbly objects. Instead of visiting these houses in person, OI caregivers used Web-based videoconferencing tools, such as Skype and FaceTime, to connect with friends and family and to keep their children in a safer environment.

###### Supporting Self-Care and Hope

Caregivers used IBTs for their own self-care and feelings of hope. Smartphone games, video streaming services, music apps, and social media for personal use were all popular tools. A common challenge for caregivers was experiencing boredom as they waited through numerous medical appointments and lengthy recoveries from surgeries. Caregivers used IBTs, such as video streaming apps and smartphone games, as distractions during these times. One mother used a relaxation app to help her focus on her breathing, which lessened her anxiety. Some caregivers used Web-based resources to find inspiration. One caregiver watched inspirational YouTube videos posted by families with OI to help *motivate* her daughter and herself (M6-III). Another mother, whose school-aged child had experienced over 150 fractures, explained:

On YouTube, I find there’s a lot of inspirational videos that are nice to watch. Experiences that turn out to be good and [show] that there’s hope.

M5-III

###### Managing the Logistics of Caregiving

Caregivers used IBTs to manage the logistics of coordinating medical appointments. There were 2 caregivers who specifically mentioned using their calendar apps to keep track of the many health care appointments that came with caring for a child with brittle bones. Caregivers generally agreed that IBTs had made accessing health care services much easier. Caregivers used emails to contact potential clinicians and make appointments, texted their social workers and physiotherapists, and sent x-ray pictures to their treating physicians. A few caregivers created their own digital medical records by compiling their emails, x-ray photos, and other documents into one digital location, such as an emails folder or a photo album.

Some families used internet tools and apps to find community and care resources. Caregivers discussed using the internet to assess an environment’s wheelchair accessibility (eg, Kijiji’s images helped them assess the accessibility of rental housing). Several families used IBTs to research products and services that could help with caring for a child with OI. One mother explained how she used the internet to research an appropriate stairlift for their home once their children were too big to be carried upstairs:

I think, yes, sometimes [the Internet] helps. Like the stairlift. We didn’t have any idea how we would do this. And then internet search[ing], we saw that all these different companies were selling these stairlifts for different price [sic]. So yeah, I think that’s helped.

M3-IV

Other examples included caregivers watching Web-based video reviews of potential wheelchairs to buy and researching OI-appropriate activities and adapted sports for their children to engage in.

Finally, caregivers used IBTs to continue managing their careers. One father, whose family travelled far to receive treatment for their 2 children with OI, explained he often used video calling to call his children after surgeries when he was unable to be physically present. This father also had to give up his business providing income tax services because it was not flexible enough to meet his family’s needs. Instead, he became a taxi driver who supplemented his income as an Uber driver. Another father explained that the internet allowed him to resolve a workplace emergency remotely while he was waiting for his daughter’s planned surgery at the hospital.

##### Facilitating Osteogenesis Imperfecta Medical Information Seeking

Caregivers noted that a common activity relating to OI care was seeking information about the condition. Caregivers were selective in how they used IBTs for finding medical information. They preferred to receive medical and prognostic information from clinicians, but they would often seek information regarding day-to-day OI care activities using Web-based resources. However, as a mother from a country with limited access to OI support explained, for some families, the internet was their only accessible source for OI information:

To me, I think [IBT is] good. It is really helping. Because if you should look at where I’m coming from...and where the kind of information that I can even access back home, I think it’s excellent. It bridges the gap. Even though we are far apart, we are able to know most of the things going on.

M6-III

For some caregivers with limited access to OI expertise, wading through OI websites found on the internet was their only option for learning about their child’s medical needs.

##### Facilitating Osteogenesis Imperfecta Social Networking

Caregivers, primarily mothers, were strategic in how they used OI social media. Most preferred social media for meeting other OI families. They shared day-to-day care information rather than using it for specific information on prognoses or treatments. Some caregivers were more interested in answering others’ questions than in having their own questions answered. Several caregivers expressed an interest in meeting and conversing with other families with OI to learn more about each other’s OI experiences, and in doing so, these caregivers formed strong connections through social media. One mother, although cautious regarding specific pieces of OI information being shared on social media, kept in touch with over 200 people from the OI community on Facebook. Another mother explained:

[The OI Parents Facebook group is] a huge, huge, huge—if not the biggest help, with [OI son] and being a caregiver for him. The parents, the families that are on there, are awesome. Everybody is just—so helpful.

M9-III

Caregivers who frequently participated on OI social networks also used these platforms for sharing practical advice with each other:

Useful tips and tricks to help after rodding, after a fracture, adjusting the wheelchair—like, those types of things.

M13-III

One mother explained that OI parents were the ones who know practical day-to-day care strategies, such as where to find adaptive clothing and winter boots that would fit her child’s physique. Web-based caregivers could receive faster answers to questions from multiple sources, such as information on how to splint a new fracture, than if they sought out input from their health care providers. For a few of these families, the first time they learned about the study hospital site being an OI treatment center was through OI social networks rather than through a health care professional.

#### Concerns of Internet-Based Technologies

Caregivers’ concerns about IBTs typically fell into 2 categories: health and screen time concerns and concerns regarding the quality of IBT content. Geographic differences in these IBT concerns were noted.

#### Health and Screen Time Concerns

Caregivers expressed concerns about the potential negative mental and physical health effects of too much screen time for themselves and for their children. One mother shared:

The OI Parents group can be really good. [But] I’ve excused myself from following it on my newsfeed because it was all I saw, all day... I found that all OI, all of the time, was making that our life. Instead of OI being a part of our life, it was taking over.

M13-III

Some caregivers were concerned about Web-based predators. Others worried about cyberbullying directed toward their children or cyber judgement from other OI parents. Although caregivers consistently expressed concerns regarding both themselves and their children spending too much time on their devices, a few explained that they were somewhat less strict with IBT use for their children with OI because they could not do as many physical activities as other children, especially during a fracture, immobilization, or a painful period.

#### Quality of the Internet-Based Technology Content

The most prominent concern regarding IBT was the quality of the content available. Several families described their disturbing experiences of trying to find information about OI on the Web. When 1 mother tried to find more information on OI through a search engine, she found the following:

Angulated limbs, like fresh fractures, bone through skin, those kinds of things, which was terrible for a postpartum mother to see about their tiny little baby. So, I have stopped [searching].

M13-III

Oftentimes, what caregivers found first on the Web were the worst-case scenarios that were not applicable to their own children.

Families preferred to speak with expert clinicians about the medical treatment and prognostic aspects of OI. Several caregivers explained that as there is so much variation in the presentation of OI, Web-based prognostic information relevant to their own cases was limited. It also cost caregivers a lot of time to determine which websites and tools had accurate high-quality information. Conflicting information and disinformation led to a few caregivers seeking clinical OI information solely from the study hospital site. They preferred to explore Web-based resources recommended from trusted health care institutions.

#### Geographic Differences in Internet-Based Technologies Concerns

Caregivers who had easier access to the study hospital site appeared to have more concerns regarding OI social networks and Web-based resources. For instance, a father who had lived with OI and near the study site most of his life explained that he probably had no interest in OI social media for 2 reasons: he had searched on social media for OI once and believed the information to be very inaccurate and because “for us, [OI is] known. And that’s the big difference” [F1-IV]. In contrast, when asked about her views on IBTs, a mother from a country with limited accessible OI care options shared:

I want anybody that has OI should have internet access. Because it gives fast access to people you’re supposed to connect [with], once you have an emergency.

M12-IV

This mother learned of OI treatment and the study hospital site through an international social media group for OI families. Before learning about this group, she felt isolated, as if she was the only parent ever who had a child with brittle bones. Through the Web-based group, other international members connected with her and sent her bisphosphonate treatments. With bisphosphonates, her school-aged daughter’s bones strengthened, and she was able to mobilize more freely, enabling her to attend school. The Web-based group also managed to connect this mother with a local sponsor so her daughter could receive surgical treatments at the study site.

#### Strategies and Suggestions for Optimizing Internet-Based Technologies

##### Advice to Other Caregivers Regarding Internet-Based Technology Strategies

Caregivers shared the IBT advice they would share with other caregivers on how to use IBTs optimally with their children and for themselves. They recommended using Web-based videos and apps for distracting their children when they are in pain or immobilized. Websites that some caregivers suggested for learning more about OI included YouTube videos about the OI experience, OI Facebook groups, and the OI Foundation and Shriners Hospitals for Children—Canada websites and social media pages. They advised that caregivers should monitor the content their children view and that caregivers should be somewhat skeptical consumers of Web-based content. They also stressed the importance of being role models to their children when using IBTs, such as by limiting parents’ and children’s screen time.

##### Suggestions for Internet-Based Technology Development

OI caregivers offered many ideas for desirable IBT products and services to better support their needs (Textbox 1). In general, caregivers desired child-friendly and age-appropriate IBTs, and they wanted these resources to be available on the Web to regularly inform caregivers about research and medical updates. Caregivers said that when a service is meant to provide answers to questions, it should be interactive and quickly responsive. Francophone families particularly emphasized that there were not enough resources in French, limiting their access to support. Some caregivers suggested they would be interested in social media resources to understand what adulthood experiences of OI are like. A few families said they had never been told about nor had they found any OI-specific IBT resources. These caregivers wanted expert OI institutions to recommend more Web-based OI resources. Caregivers stated that resources should be offered in multiple languages, with Web-based and offline capabilities, with geographically specific listings, and in both short summaries and more extensive reports or videos.

**Internet-based technologies that caregivers would like to see developed to facilitate caregiving.**

Internet-based technologies (IBTs) to facilitate daily activities related to caregiving and logistics:

- An email coordinating service that sends caregivers’ emailed questions to the correct medical department or contact
- The option to schedule appointments on the Web
- A Web-based platform informing caregivers of relevant government financial aids and services
- The option to have videoconferencing consultations with clinicians

IBTs to verify medical information:

- Web-based, regularly updated information on the gold standards of care and treatment for osteogenesis imperfecta (OI)
- Downloadable software for OI families with descriptions of the condition, available resources, new treatments, and recent research updates
- Web-based home fracture-splinting videos with diverse techniques for every fracture possible
- Web-based videos portraying OI patients before and after various treatments
- A Web-based listing of a group of trusted parents and clinicians who caregivers can contact
- A gamified app to teach children occupational therapy and physiotherapy

IBTs to engage in social networking and share experiences with OI families:

- An OI social network where parents can share practical knowledge and videos of caregiving techniques with each other
- A way to organize the OI Parents Facebook group content so that the information it contains is easier to navigate
- A website to donate medical equipment, such as wheelchairs, to other families

## Discussion

### Principal Findings

We found that OI caregivers were using IBTs to care for themselves and for their children with OI. Yet, although caregivers generally held positive views of IBTs, they were also concerned about the potential negative health effects of IBTs and the quality of IBT content. Caregivers offered suggestions for how other OI caregivers could optimize the use of IBTs in their caregiving lives and for how clinicians and software developers could build better IBTs to support caregivers.

Caring for a child with OI may lead to physical, relational, and self-care challenges [6,22]. Our study found that caregivers were facing similar challenges, but it also revealed the benefits of using IBTs to manage these challenges. For example, OI caregivers used IBTs as a distraction to manage the physical challenges associated with treating their child’s pain. Social challenges, such as social isolation from family members and friends, were being partially overcome through videoconferencing support. Caregivers’ physical, emotional, and self-care challenges were being addressed through IBT support. New IBT platforms may also target these needs.

Researchers have described the many logistical challenges of caring for a child with OI, such as coordinating appointments, finding resources, and communicating with clinicians [6,7]. These logistical challenges were also noted in this study. Smartphone apps are presently being developed for caregivers of other chronically ill children to communicate with other family members and health care teams to coordinate care [23-25]. Other potential platforms for addressing these logistical challenges include teleconferencing with clinicians and Web-based appointment scheduling [13]. Similar apps may be developed to support OI caregivers.

Several caregivers in this study experienced difficulties in finding health care providers who were knowledgeable about OI. These difficulties have been previously reported by OI caregivers [6,22]. For medical information on OI, such as treatments and fracture prognoses, caregivers much preferred information that came from a knowledgeable OI health care provider or institution. However, when knowledgeable providers were not available, caregivers turned to the internet. This is a similar strategy used by other rare disease populations to fulfill their information needs [12,14]. Web-based resources serve as sources of information and support, particularly when specialists are not accessible [12-14].

Caregivers’ views on IBTs for social and medical information seeking appeared to depend, in part, on their relative access to the study site’s OI specialist center: if caregivers did not have easy access to OI medical specialists (eg, because of geographical constraints), they relied more on Web-based OI resources and communities. This pattern corresponds with research suggesting caregivers of children with special needs have more of an impetus to seek information on the Web than do caregivers of children without special needs [14,15]. This pattern suggests that IBTs may be most useful for caregivers who have internet access but do not have regular or easy access to experts. Still, similar to the OI caregivers in our study, many caregivers have concerns regarding the health effects and quality of the content of IBTs [14,26]. Leading OI institutions should work to develop and share Web-based services, ensuring they provide high-quality information and content.

### Clinical and Research Implications

Clinicians should inquire about families’ current uses of IBTs and help link families to other credible sources [12,27]. The study site’s recent adoption of the *Upopolis* platform, which helps children with medical support needs find information and connect with other friends and families, is an example of a credible existing tool being shared by clinicians [28]. Families, clinicians, and researchers should work together to create resources using participative approaches. Using participatory design approaches to build new interventions increases the likelihood that end users will accept the interventions when they are launched [16,29]. Recent examples in OI include the creation of the Good2Go MyHealth Passport, a tool for optimizing the transfer of pediatric patients to adult health care services and prototype development of a tool to engage children with OI in their care [30-32]. OI caregivers’ suggestions and strategies for supportive IBTs should be used to improve clinical practice and to develop future IBT services for families with OI [33].

### Study Limitations and Strengths

Our study has several limitations. Regarding our data collection and analysis methods, ideally for the 6 French interviews, we would have independently back translated the English translations into French to ensure that the translations were trustworthy [34]. Owing to time and resource limitations, we did not do this, as 5 of the 6 team members and coauthors were fluently bilingual. One of the coauthors, KC, is fluently bilingual in English and French and has a rich personal knowledge of the subject material [34]. She was responsible for open coding all the French interviews into English. Regarding our sample, this study was conducted at an internationally renowned treatment center for OI. Therefore, we were conducting interviews with caregivers who had the resources to access this center. The demographic survey data revealed that most participants were financially well off and had at least some postsecondary education. Future research should work to explore the IBT views and needs of OI caregivers who do not have access to international treatment centers and who have even more diverse socioeconomic backgrounds than these caregivers did.

Strengths of the study design include a rich description of the setting and of the participants. These data add context to the study, helping the reader to decide if and whether the results and interpretations are relevant to their own situation or research [35]. The interviews were comprehensive (lasting between 15 min and 1.5 hours), and the sampling was purposive to include a range of OI caregiver experiences, creating a deep dataset for analysis. Finally, as far as we are aware, this is the first study to research in depth how caregivers of children with OI view and use IBTs to support their caregiving activities. Our research offers a starting point for future researchers to build more supportive caregiving technologies.

### Conclusions

Through this study, we have a better understanding of how OI caregivers can use specific IBTs to facilitate daily activities, information seeking, and social networking in relation to OI. We also have a better understanding of their concerns regarding IBTs and how future IBT projects may be built to optimize the benefits of IBTs while reducing the concerning aspects of IBTs. Clinicians should share the strategies that OI caregivers recommend for optimizing IBT use in caregiving with families newly diagnosed with OI. Some of these strategies may also be useful for other caregivers caring for youths with rare or chronic conditions. Clinicians should work toward developing IBTs that meet the needs and suggestions of the caregiver participants in this study so that the benefits of IBTs may be realized for more caregivers, while diminishing the potential harms of IBTs.

## Appendix

Multimedia Appendix 1 Interview guide for interviews with caregivers who identified as internet-based technology users.

## Abbreviations

IBT: internet-based technology
OI: osteogenesis imperfecta

## References

[1] Marini JC, Forlino A, Bächinger HP, Bishop NJ, Byers PH, Paepe AD, Fassier F, Fratzl-Zelman N, Kozloff KM, Krakow D, Montpetit K, Semler O (2017). Osteogenesis imperfecta. Nat Rev Dis Primers.

[2] Trejo P, Rauch F (2016). Osteogenesis imperfecta in children and adolescents-new developments in diagnosis and treatment. Osteoporos Int.

[3] Dogba MJ, Bedos C, Durigova M, Montpetit K, Wong T, Glorieux FH, Rauch F (2013). The impact of severe osteogenesis imperfecta on the lives of young patients and their parents - a qualitative analysis. BMC Pediatr.

[4] Hill CL, Baird WO, Walters SJ (2014). Quality of life in children and adolescents with Osteogenesis Imperfecta: a qualitative interview based study. Health Qual Life Outcomes.

[5] Hu C, Kung S, Rummans TA, Clark MM, Lapid MI (2015). Reducing caregiver stress with internet-based interventions: a systematic review of open-label and randomized controlled trials. J Am Med Inform Assoc.

[6] Arabaci LB, Bozkurt S, Vara S, Ozen S, Darcan S, Simsek DG (2015). Difficulties experienced by caregivers of patients diagnosed with osteogenesisimperfecta (OI): example of a hospital. J Pak Med Assoc.

[7] Claesson IB, Brodin J (2002). What families with children with brittle bones want to tell. Child Care Health Dev.

[8] Bozkurt S, Arabacı LB, Vara S, Ozen S, Gökşen D, Darcan S (2014). The impact of psycho-educational training on the psychosocial adjustment of caregivers of osteogenesis imperfecta patients. J Clin Res Pediatr Endocrinol.

[9] Drake TM, Ritchie JE (2016). The surgeon will Skype you now: advancements in e-clinic. Ann Surg.

[10] Girault A, Ferrua M, Lalloué B, Sicotte C, Fourcade A, Yatim F, Hébert G, di Palma M, Minvielle E (2015). Internet-based technologies to improve cancer care coordination: current use and attitudes among cancer patients. Eur J Cancer.

[11] González-Herrero A, Smith S (2008). Crisis communications management on the web: how internet-based technologies are changing the way public relations professionals handle business crises. J Conting Crisis Manag.

[12] Naftel RP, Safiano NA, Falola MI, Shannon CN, Wellons JC, Johnston JM (2013). Technology preferences among caregivers of children with hydrocephalus. J Neurosurg Pediatr.

[13] Tozzi AE, Mingarelli R, Agricola E, Gonfiantini M, Pandolfi E, Carloni E, Gesualdo F, Dallapiccola B (2013). The internet user profile of Italian families of patients with rare diseases: a web survey. Orphanet J Rare Dis.

[14] Knapp C, Madden V, Wang H, Sloyer P, Shenkman E (2011). Internet use and eHealth literacy of low-income parents whose children have special health care needs. J Med Internet Res.

[15] DeHoff BA, Staten LK, Rodgers RC, Denne SC (2016). The role of online social support in supporting and educating parents of young children with special health care needs in the united states: A scoping review. J Med Internet Res.

[16] Matthew-Maich N, Harris L, Ploeg J, Markle-Reid M, Valaitis R, Ibrahim S, Gafni A, Isaacs S (2016). Designing, implementing, and evaluating mobile health technologies for managing chronic conditions in older adults: a scoping review. JMIR Mhealth Uhealth.

[17] Sandelowski M (2000). Whatever happened to qualitative description?. Res Nurs Health.

[18] Braun V, Clarke V (2006). Using thematic analysis in psychology. Qual Res Psychol.

[19] DeCuir-Gunby JT, Marshall PL, McCulloch AW (2011). Developing and using a codebook for the analysis of interview data: an example from a professional development research project. Field Methods.

[20] Guba EG, Lincoln YS (1985). Naturalistic Inquiry.

[21] Polit DF, Beck CT (2012). Nursing Research: Generating And Assessing Evidence For Nursing Practice. Ninth Edition.

[22] Dogba MJ, Rauch F, Tre G, Glorieux FH, Bedos C (2014). Shaping and managing the course of a child's disease: parental experiences with osteogenesis imperfecta. Disabil Health J.

[23] Mueller EL, Cochrane AR, Bennett WE, Carroll AE (2018). A survey of mobile technology usage and desires by caregivers of children with cancer. Pediatr Blood Cancer.

[24] Wang J, Yao N, Wang Y, Zhou F, Liu Y, Geng Z, Yuan C (2016). Developing 'Care Assistant': a smartphone application to support caregivers of children with acute lymphoblastic leukaemia. J Telemed Telecare.

[25] Liu X, Wang R, Zhou D, Hong Z (2016). Smartphone applications for seizure care and management in children and adolescents with epilepsy: Feasibility and acceptability assessment among caregivers in China. Epilepsy Res.

[26] Radesky JS, Kistin C, Eisenberg S, Gross J, Block G, Zuckerman B, Silverstein M (2016). Parent perspectives on their mobile technology use: the excitement and exhaustion of parenting while connected. J Dev Behav Pediatr.

[27] Christensen S, Morelli R (2012). MyPapp: a mobile app to enhance understanding of pap testing. Comput Inform Nurs.

[28] (2017). Kids' Health Links Foundation.

[29] Clemensen J, Larsen SB, Kyng M, Kirkevold M (2007). Participatory design in health sciences: Using cooperative experimental methods in developing health services and computer technology. Qual Health Res.

[30] Jeong S, Chougui K, Mercier C, Wong T, Lafrance M, Gagnon V, Plourde S, Rauch F, Bilodeau C, Thorstad K, Tsimicalis A (2019). Development of the Good2Go MyHealth Passport for individuals with Osteogenesis Imperfecta: a knowledge-synthesis study. Int J Orthop Trauma Nurs.

[31] Carrier JI, Siedlikowski M, Chougui K, Plourde S, Mercier C, Thevasagayam G, Lafrance M, Wong T, Bilodeau C, Michalovic A, Thorstad K, Rauch F, Tsimicalis A (2018). A best practice initiative to optimize transfer of young adults with osteogenesis imperfecta from child to adult healthcare services. Clin Nurse Spec.

[32] Siedlikowski M, Rauch F, Ruland C, Tsimicalis A (2018). Giving children a voice: development of an interactive assessment and communication tool for osteogenesis imperfecta. J Pain Symptom Manage.

[33] Dogba MJ, Dahan-Oliel N, Snider L, Glorieux FH, Durigova M, Palomo T, Cordey M, Bédard MH, Bedos C, Rauch F (2016). Involving families with osteogenesis imperfecta in health service research: joint development of the OI/ECE questionnaire. PLoS One.

[34] Chen HY, Boore JR (2010). Translation and back-translation in qualitative nursing research: methodological review. J Clin Nurs.

[35] Tong A, Sainsbury P, Craig J (2007). Consolidated criteria for reporting qualitative research (COREQ): a 32-item checklist for interviews and focus groups. Int J Qual Health Care.

