# Supplementary material for: Exploring the Views of Osteogenesis Imperfecta Caregivers on Internet-Based Technologies: Qualitative Descriptive Study
Source: J Med Internet Res. 2019 Dec 18;21(12):e15924. doi: 10.2196/15924 (PMC6939279; doi:10.2196/15924)
Supplement: Multimedia Appendix 1 [file jmir_v21i12e15924_app1.docx]

Multimedia Appendix 1: Interview guide for interviews with caregivers who identified as IBT users.

| 1. Before we begin, would you mind filling out this demographic survey, please? We ask these questions so that we have a better understanding of each OI caregiver’s unique situation. *[Participant fills in survey.]* 2. I have not looked after a child who has OI. Can you describe what a regular day looks like when caring for your child with OI? And then can you describe a more challenging day recently? 3. Throughout today’s interview, I will be referring back to “IBT” or “Internet-based technologies.” These are... [*Explain further using storyboard poster with the different examples of IBT {social media, email, messenger apps, relaxation apps, tablet computers, etc.}].* To reiterate, we are interested not only in technologies that you use when directly providing care for your child, but also in tools that you use in your personal, day-to-day life. 4. Based on this description of IBT, generally would you consider yourself to be someone who uses the Internet and/or Internet applications in your daily life?   *[If “yes” proceed with this interview guide; if “no,” switch to interview guide 2.]*   1. What do you think about using the Internet and mobile apps in your daily care for ____[child’s name]_____? 2. What suggestions might you have for how the Internet and/or smartphones could improve caring for your child and/or yourself? 3. Do you have any concerns about IBT? If so, please explain. 4. If you were giving advice to someone else who has a child with OI about using IBT in caregiving, what would you say? 5. Is there anything else you would like to discuss regarding the Internet, computers, or mobile applications; and caring for ___[child’s name]___?   Thank you very much for your time. If you know of any other OI caregivers who might be interested in this study, please feel free to give them our contact information. |
| --- |
